# Publisher Correction: Accuracy and data efficiency in deep learning models of protein expression

**DOI:** 10.1038/s41467-023-38752-7

**Published:** 2023-05-18

**Authors:** Evangelos-Marios Nikolados, Arin Wongprommoon, Oisin Mac Aodha, Guillaume Cambray, Diego A. Oyarzún

**Affiliations:** 1 School of Biological Sciences, University of Edinburgh, Edinburgh, EH9 3JH UK; 2 School of Informatics, University of Edinburgh, Edinburgh, EH8 9AB UK; 3 The Alan Turing Institute, London, NW1 2DB UK; 4 Diversité des Génomes et Interactions Microorganismes Insectes, University of Montpellier, INRAE UMR 1333, Montpellier, France; 5 Centre de Biologie Structurale, University of Montpellier, INSERM U1054, CNRS UMR5048, Montpellier, France

**Keywords:** Synthetic biology, Machine learning

Correction to: *Nature Communications* 10.1038/s41467-022-34902-5, published online 15 December 2022

The original version of this Article contained an error in Fig. 2, in which the bottom captions for the heatmap in Fig. 2b were inadvertently shifted downward into Fig. 2c. This has been corrected in both the PDF and HTML versions of the Article.

