# Supplementary material for: Accuracy and data efficiency in deep learning models of protein expression
Source: Nat Commun. 2022 Dec 15;13:7755. doi: 10.1038/s41467-022-34902-5 (PMC9751117; doi:10.1038/s41467-022-34902-5)
Supplement: Supplementary file 1 — Supplementary Information [file 41467_2022_34902_MOESM1_ESM.pdf]

Supplementary Text

Accuracy and data efficiency in deep learning models of protein expression

Evangelos-Marios Nikolados[1], Arin Wongprommoon[1], Oisin Mac Aodha[2,3], Guillaume Cambray[4], Diego A. Oyarzún[1,2,3†]

[1] School of Biological Sciences, University of Edinburgh, Edinburgh EH9 3JH, UK

[2] School of Informatics, University of Edinburgh, Edinburgh EH8 9AB, UK

[3] The Alan Turing Institute, London, NW1 2DB, UK

[4] DGIMI, Université Montpellier, INRA, Montpellier, France.

† Corresponding author d.oyarzun@ed.ac.uk

Introduction

Here, we detail the promoter dataset in *Saccharomyces cerevisiae* from Vaishnav et al[1]. In the original work, this dataset was employed as a test set to demonstrate the generalization performance of a model trained on ∼20M promoter sequences; these results are shown in Supplementary Figure 4F of Vaishnav et al[1]. We repurposed this dataset to train sequence-to-expression models with a reduced number of variants and test our results on sequence diversity (Figure 5 in main text) in a different expression host and construct library.

Dataset

The dataset contains expression levels of 3,929 promoter sequences as measured by a large parallel reporter assay[2], in which 80bp sequences were embedded within a promoter construct and the expression of a YFP reporter was assayed in a *S. cerevisiae* strain lacking URA3. The library consists of native yeast promoter sequences from 199 genes, each one with an average of 20 random single base mutations. Constructs were cloned within the -160:-80 region, relative to the transcription start site of a synthetic promoter scaffold, a critical location for transcription factor binding[3] and determinant of promoter activity[2]. The promoter construct was placed in a dual reporter plasmid that contains URA3 (used as a selectable marker), a constitutive RFP reported (to control for extrinsic noise), and the YFP reporter under variable control. Finally, yeast cells were

cultivated in synthetic defined medium lacking uracil (SD-Ura), sorted into 18 uniformly sized expression bins. Promoters in each bin were sequenced to estimate YFP expression level.

For ease of use by the community, we have cleaned and organized the dataset in a form suitable for machine learning work. The cleaned dataset can be found in the Zenodo repository at `https://doi.org/10.5281/zenodo.7273952`.

Impact of sequence diversity on model accuracy

Strategy For the results in Figure 6, we first aggregated the variant clusters into twelve groups, with each group containing variants from ∼16 randomly selected clusters. We then trained regressors on group aggregates, in a similar fashion to the analysis in Figure 5. The models in Figure 6 were trained on datasets of constant size and increasing sequence diversity. We successively aggregated fractions of groups to create new training sets with improved diversity.

Given the small size of the training data (∼330 sequences/group), we fixed the size of the training set to 400 sequences and focused on training Random Forest models. To increase diversity, for successive models we sampled training sequences from two additional groups, as shown in Figure 6B. The specific groups for the aggregates were randomly chosen; four training repeats with randomized selection of groups can be found in Supplementary Figure S13.

Training, validation and test data To ensure a balanced held-out test set, we uniformly sampled 20% of the 20 sequences that include point mutations for each of the 199 native genes present in the yeast dataset. This resulted in 588 held-out sequences that we reserved for testing all downstream random forest models. The remaining 80% of the total dataset was further partitioned in a similar manner to acquire 780 sequences that we used as a fixed validation set for hyperparameter optimization and 2560 sequences that we used for training.

Hyperparameter selection We performed hyperparameter optimization using binary one-hot encoding and the 780 sequences in the validation set. Hyperparameters were determined via grid search with 10-fold cross-validation. The hyperparameter search space and the resulting random forest configuration, used for all models trained on the yeast dataset, can be found in Supplementary Table S6. We employed the same hyperparameters for all five models in Figure 6B in the main text.

Supplementary Figures

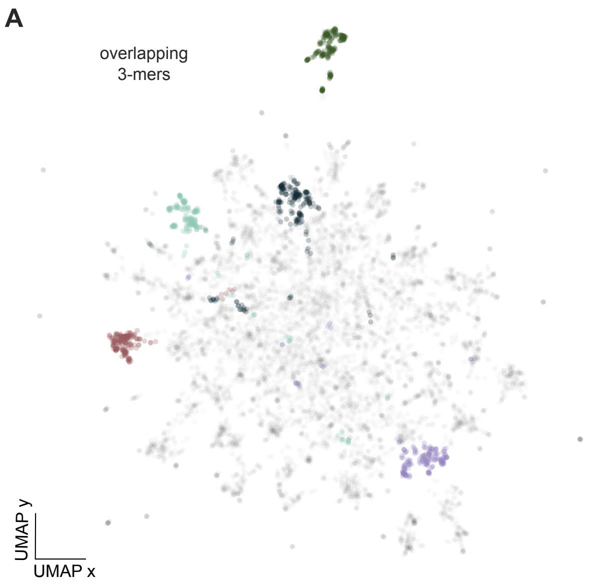

Supplementary Figure S1. **Two dimensional projections of the 228,000 sequences employed for training. (A)** UMAP projection for overlapping 3-mers; the choice of 3-mers does not have enough granularity for UMAP to distinguish between mutational series. **(B)** UMAP projection for overlapping 5-mers, which show a similar cluster structure as the one computed with 4-mers in Figure 1B. Colour coding is the same as in Figure 1B.

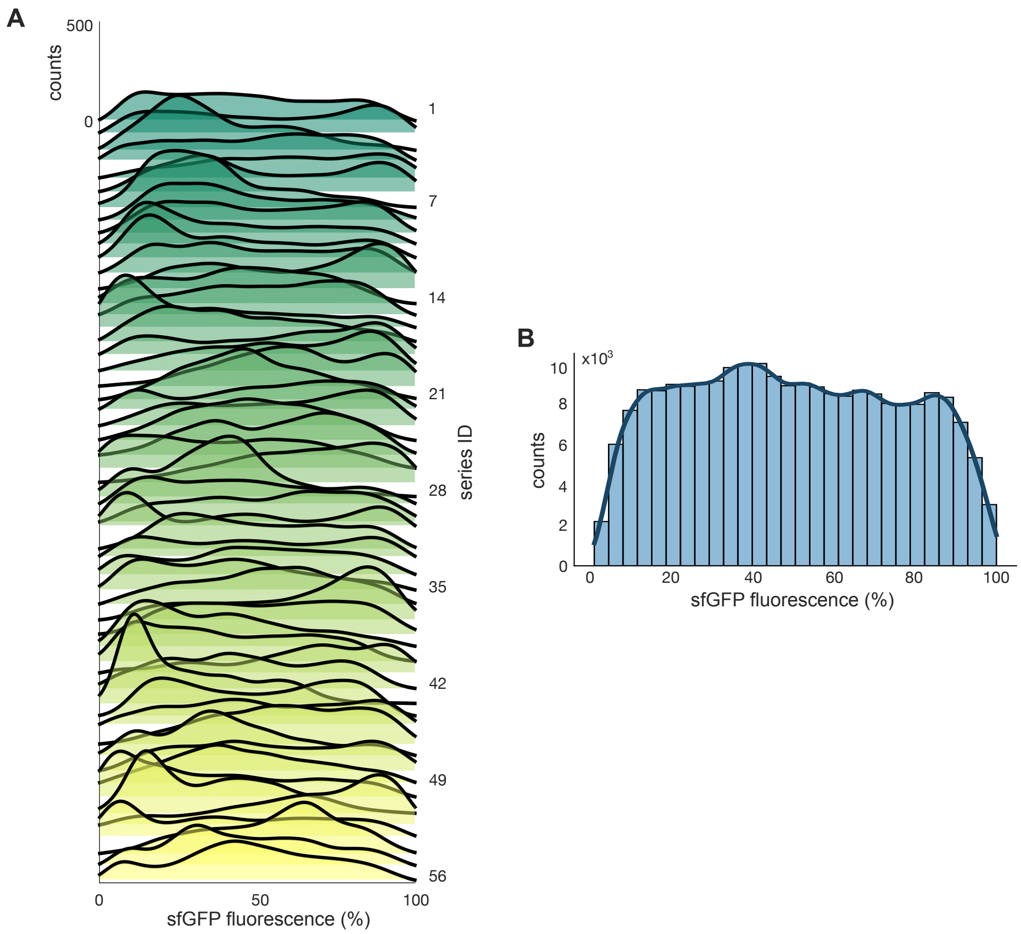

Supplementary Figure S2. **Distributions of sfGFP fluorescence from the dataset in Cambray et al[4]. (A)** Phenotypic distributions for each of the 56 mutational series. **(B)** Phenotypic distribution of the complete dataset with 56 mutational series and ∼228,000 sequence variants. Shown distributions are Gaussian kernel density estimates of fluorescence measurements averaged across four experimental replicates. Measurements are normalized to the maximum sfGFP fluorescence in the whole library.

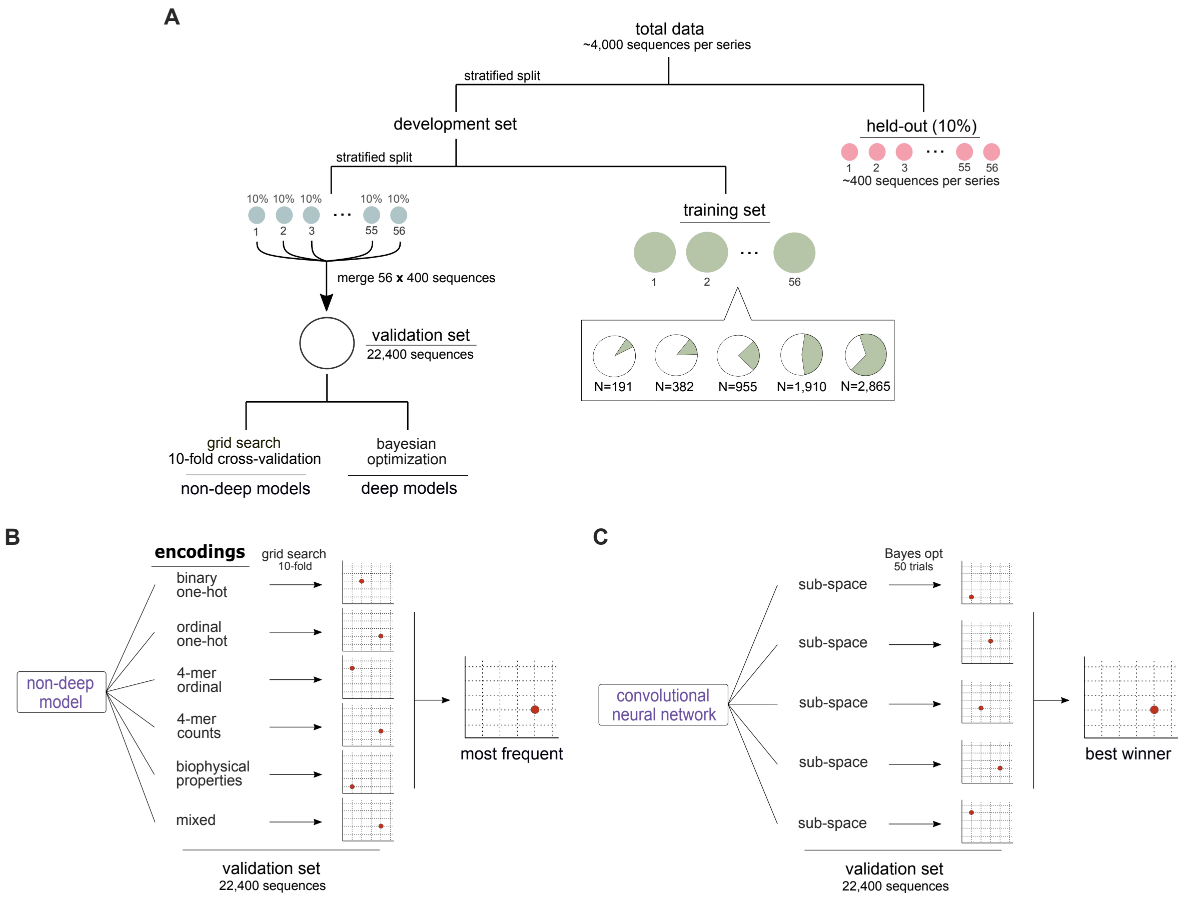

Supplementary Figure S3. **Data splitting and hyperparameter optimization strategy. (A)** Schematic of data partitioning into separate sets for training, validation and testing. We first held-out 10% of each series (∼400 sequences) for model testing; none of these sequences were used for training or cross-validation. The remaining sequences were further partitioned into a training set (employed to train models on varying data sizes) and a large validation set with 22,400 sequences from all mutational series. The validation set was employed to determine hyperparameters with 10-fold cross-validation (non-deep models) and Bayesian optimization (deep models). **(B)** Hyperparameter tuning for non-deep models. We explored the hyper-parameter space (Supplementary Table S2) using grid search and 10-fold cross-validation for each DNA encoding on 90% of the full validation set. This resulted in one configuration per encoding, from which we selected the most frequent configuration among the six encodings. For cases without a single most frequent configuration, we selected the one with the smallest mean squared error (MSE) on the remaining 10% of the validation set. **(C)** Hyperparameter tuning for CNN models. We ran five iterations of Bayesian optimization implemented in the HyperOpt package (Supplementary Table S4) and obtained five candidate architectures, from which we settled on the one with smallest MSE on the remaining 10% of the validation set.

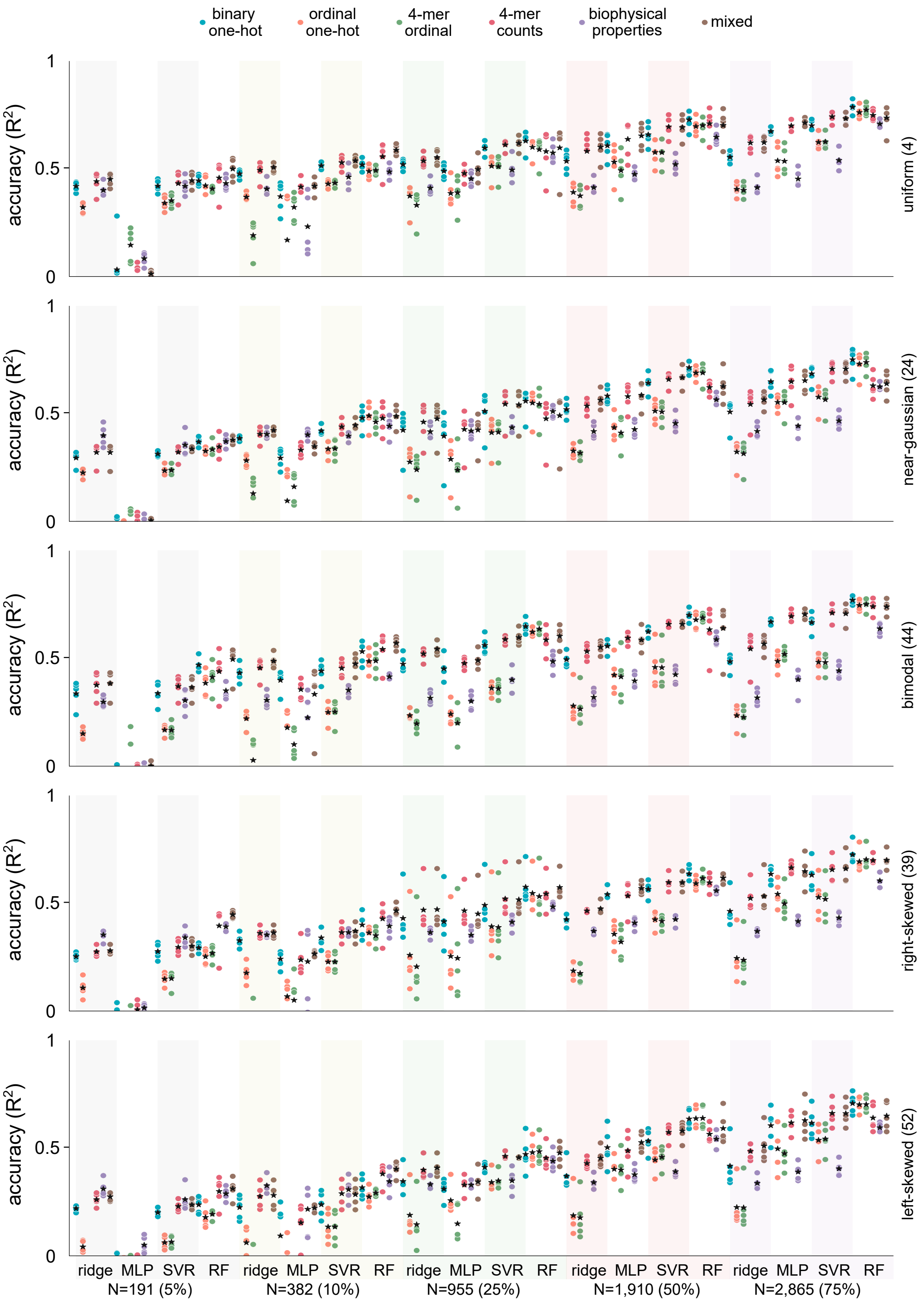

Supplementary Figure S4. **Cross-validation of non-deep models in Figure 2B**. Shown are the R^2 scores computed between measured and predicted fluorescence in held-out sets of sequences; dots are the R^2 scores for each training repeat and stars denote the mean accuracy across the five training repeats (Monte Carlo cross-validation); the plots show 600 models in total (i.e. 4 regressors $\times$ 5 data sizes $\times$ 6 encodings $\times$ 5 mutational series). In each training repeat, we held-out 10% of randomly chosen variants in each mutational series, and trained all models on the specified number of samples (N); note that in each training repeat all models were tested on the same set of held-out sequences to ensure fair comparisons. Results show robust accuracy, particularly for the high-accuracy models. There is some variation in R^2 values for low and mid accuracy regressors, but the best performing models show little evidence of overfitting.

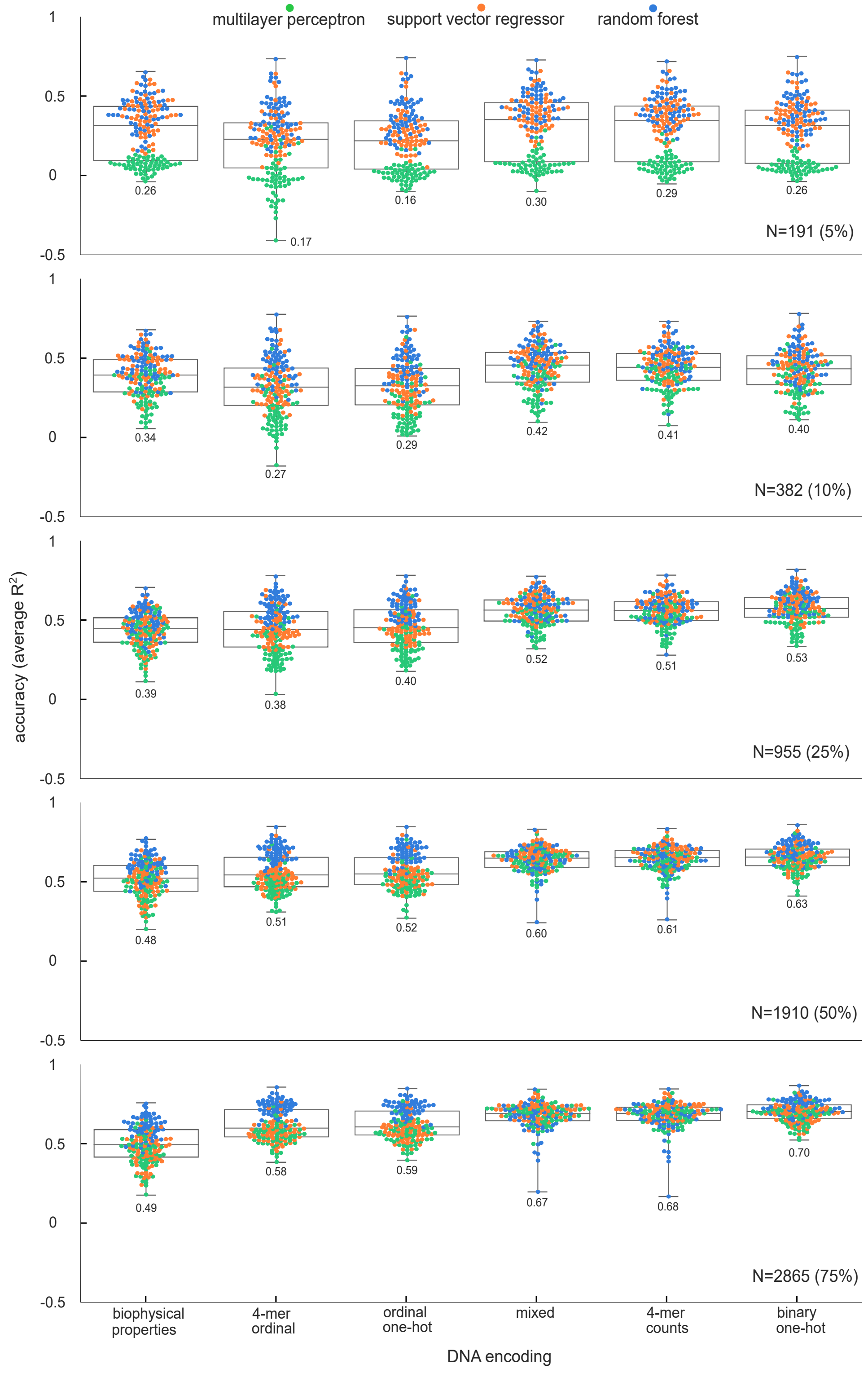

Supplementary Figure S5. **Accuracy of non-deep models trained on the whole sequence space.** We trained 5,040 models (3 regressors × 5 data sizes × 6 encodings × 56 mutational series); we excluded the ridge regressor due to its poor performance (Figure 2B). Dots in the swarm plots are the prediction accuracy scores for each model, computed as the R^2 on a fixed held-out dataset with 10% of sequences of each series, and averaged across 5 training repeats. Random forests with binary one-hot encoding provide the best accuracy. Binary one-hot encoding also lead to more consistent accuracy across series, as reflected by narrower distributions of R^2 values. For all box-and-whisker plots, the horizontal line indicates the median, box edges are at the 25th and 75th percentiles, and whiskers indicate the 1.5×IQR.

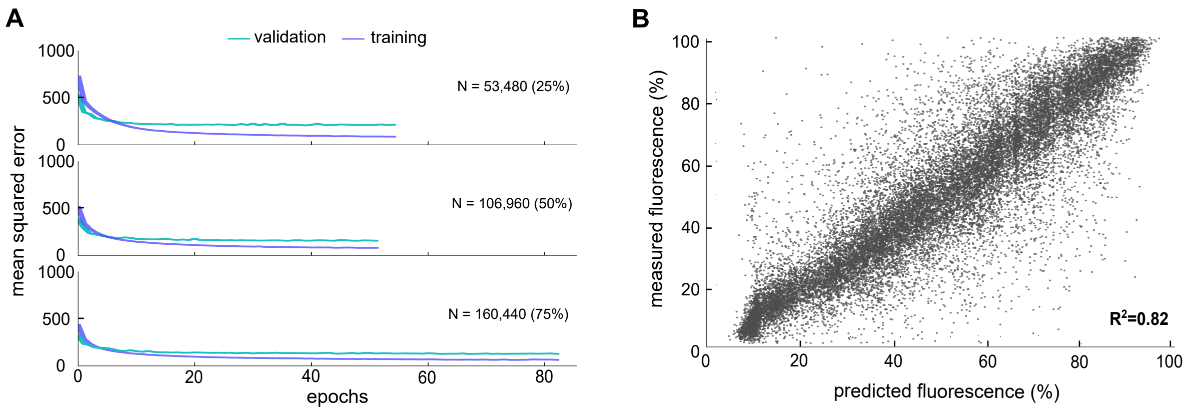

Supplementary Figure S6. **Convolutional neural network trained on all mutational series**. **(A)** Learning curves computed as the mean squared error against training epoch for the validation (cyan) and training (purple) sets for CNNs trained on 25%, 50%, and 75% of all sequences (Figure 3B). In all cases, we use the same validation set, containing 22,400 sequences aggregated over all 56 mutational series (Supplementary Figure S3A), and use 15 epochs without loss improvement on the validation set as early stopping criterion to prevent overfitting. **(B)** Predictions of the CNN from Figure 3B trained on 75% of all sequences and evaluated on held-out sequences (10% of total sequences). We note that although $R^2 = 0.82$ is comparable to the random forests models in Figure 2B, those models were trained and tested on a single mutational series; in contrast, the CNN produces accurate predictions across all mutational series.

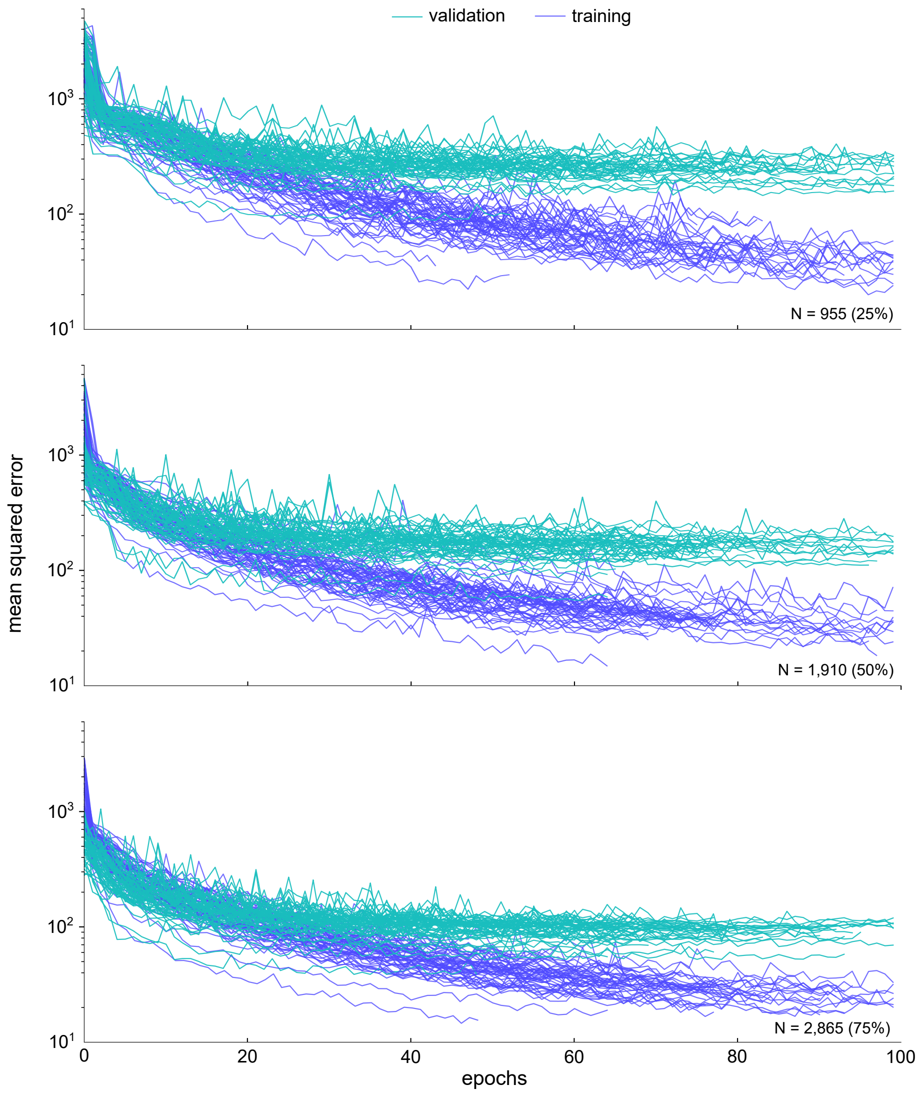

Supplementary Figure S7. **Learning curves for convolutional neural networks trained on each mutational series**. Plots show the validation (cyan) and training (purple) mean squared error against training epochs for individual CNNs trained on 25%, 50%, and 75% of the sequences in each mutational series. We use fixed validation sets with 10% of sequences from each series, and 15 epochs without loss improvement over the validation set as early stopping criterion to prevent overfitting.

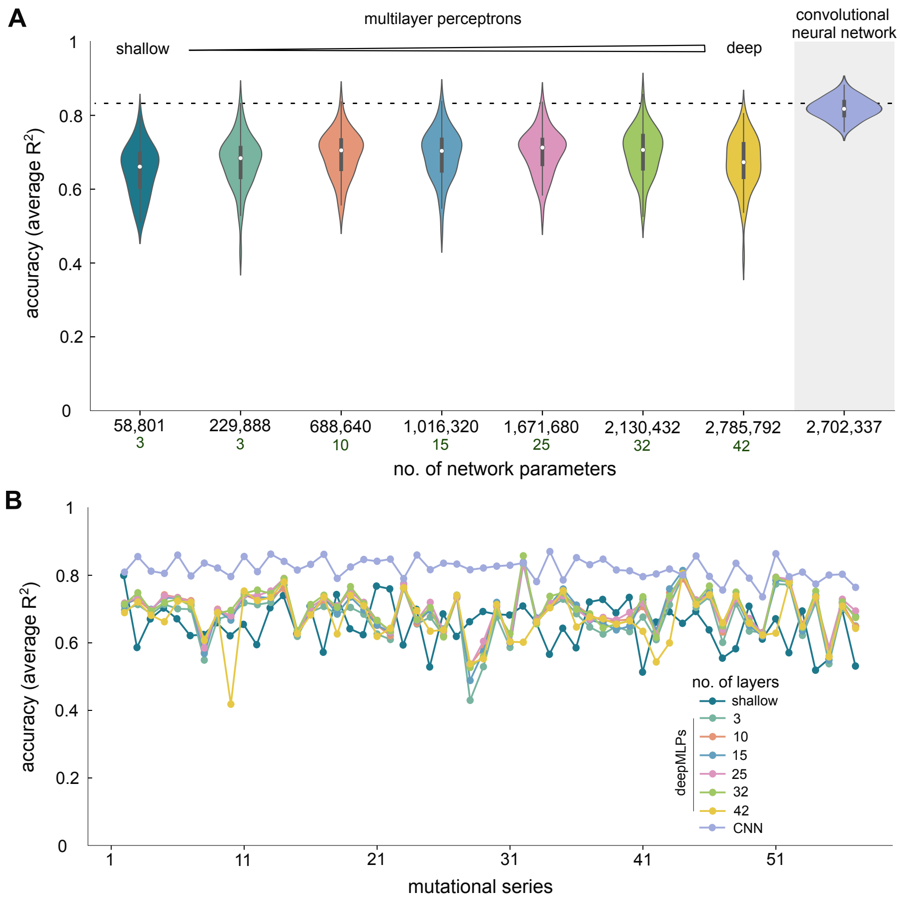

Supplementary Figure S8. **Performance comparison between deep MLPs and CNNs**. (**A**) Prediction accuracy of MLPs of increasing depth against the CNNs in Figure 3C for each of the 56 mutational series using binary one-hot encoding and 75% of sequences for training. The shallow and the first deep MLP have the same number of layers, but different number of neurons per layer, with 100 versus 256 neurons respectively. The remaining deep MLPs contain 256 neurons per layer, and were implemented in scikit-learn. The CNNs outperformed the deep MLPs even in cases when they have a similar number of trainable parameters (>2.7M parameters). This suggests that the convolutional layers can extract sequence features that are highly informative for regressing the protein expression level. Violin plots show the distribution of the 56 R^2 scores for each model averaged across 5 training repeats; R^2 values were computed on held-out sequences (10% of sequences per series). For all violins, the white circle indicate the median, box edges are at the 25th and 75th percentiles, and whiskers show 95% confidence interval. Deep MLPs were trained with the ReLU activation function and mean squared error as loss function, learning rate 1×10^{-3}, and using the Adam optimizer[5]. To prevent overfitting, we set the maximum number of epochs to 120 and used 15 epochs without loss improvement over the validation set as early stopping criterion. **(B)** R^2 scores averaged across five training repeats for each model in panel A. The deep MLPs marginally outperform the CNNs in only two mutational series (no. 32 and 43).

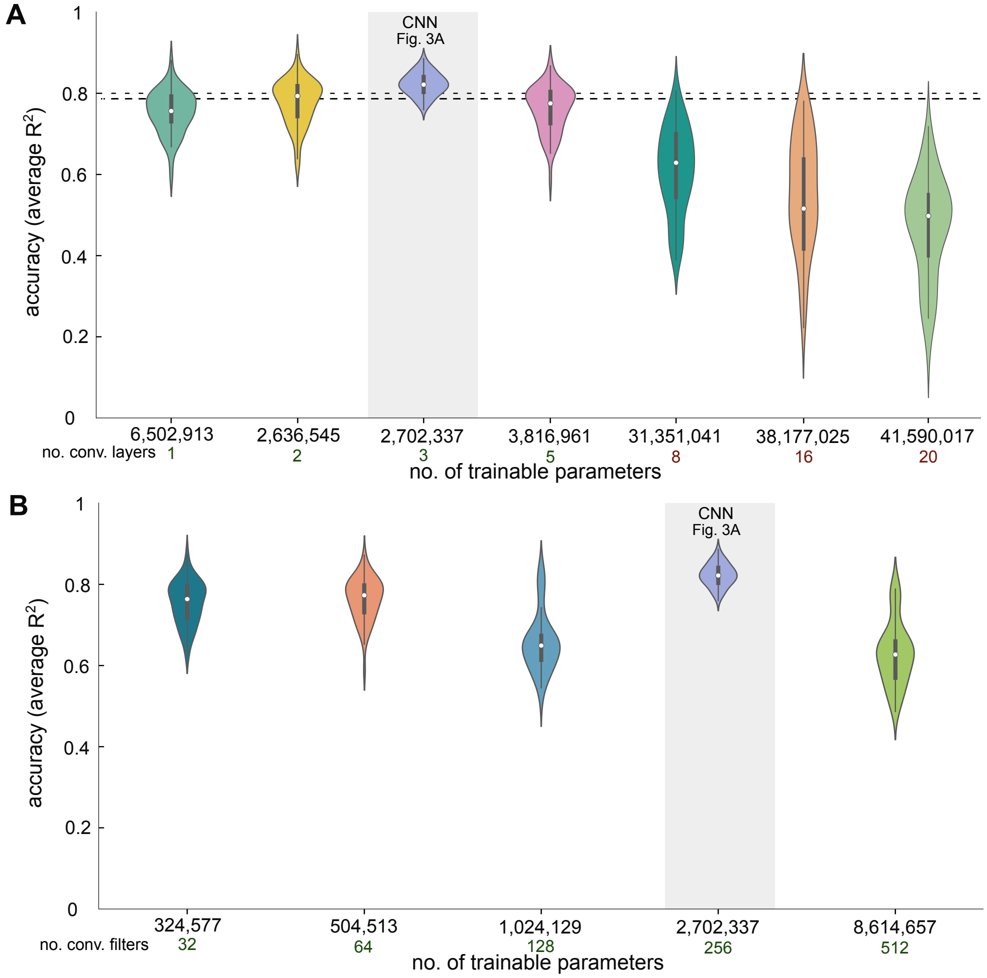

Supplementary Figure S9. **Perturbation analysis of CNNs in Figure 3A.** (**A**) Accuracy of retrained CNNs with a variable number of convolutional layers, against the CNN in Figure 3A for each of the 56 mutational series using binary one-hot encoding and 75% of sequences for training. We varied the number of convolutional layers and froze all other hyperparameters to the values in Supplementary Table S4. Violin plots show the distribution of the 56 R^2 scores for each mutational series computed on held-out sequences (10% of sequences per series) and averaged across 5 training repeats; for the deeper CNNs with $\{8, 16, 20\}$ convolutional layers we used only one training repeat. The CNN with one convolutional layer has a larger number of trainable parameters because of the lack of a max pooling layer. In CNN architectures with few convolutional layers, most of the parameters are concentrated in the dense layers. After flattening, the inputs to the dense layers are higher-dimensional than for deeper CNNs. Note that for the deeper CNNs with $\{8, 16, 20\}$ convolutional layers, we removed max pooling layers and included batch normalization to stabilize the learning process. **(B)** Prediction accuracy of CNNs with varying width (i.e. number of filters per layer) against the CNN in Figure 3A for each of the 56 mutational series using binary one-hot encoding and 75% of sequences for training. To implement CNNs of varying widths, we retrained CNNs with a variable number of convolutional filters; all other CNN hyperparameters were frozen to the values in Supplementary Table S4. In line with expectation, in panels A and B we found that the Bayesian optimized architecture (gray band, shown in Figure 3A) outperforms the other architectures. In panel B, violin plots show the distribution of the 56 R^2 scores for each mutational series computed on held-out sequences (10% of sequences per series) and averaged across 5 training repeats. For all violins, the white circle indicate the median, box edges are at the 25th and 75th percentiles, and whiskers show 95% confidence interval.

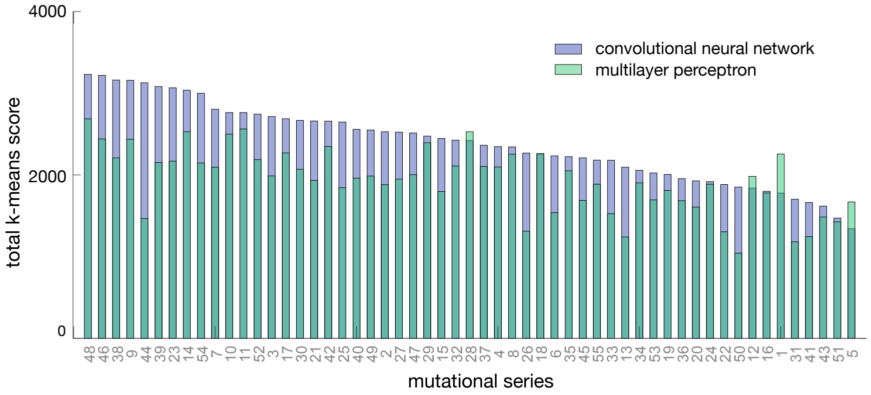

Supplementary Figure S10. **Comparison of neural networks using DeepLIFT scores**. We performed k-means clustering on the attribution distance matrices (Figure 4B) for each of the 56 models. Bars show the total k-means score for test sets in each of the CNNs shown in Figure 3C, averaged across 20 runs of the k-means clustering algorithm; the total score is defined as $\sum_{i=1}^{20} s_i$ where s_i is the clustering score for a fixed number of clusters k. Clusters were computed using DeepLIFT[6] scores as feature vectors. The CNNs display higher k-means scores in all but four mutational series, which suggests that they are better at discriminating between similar sequences in a test set; all test sets contain 10% of sequences of each mutational series ($\sim$450 sequences/series).

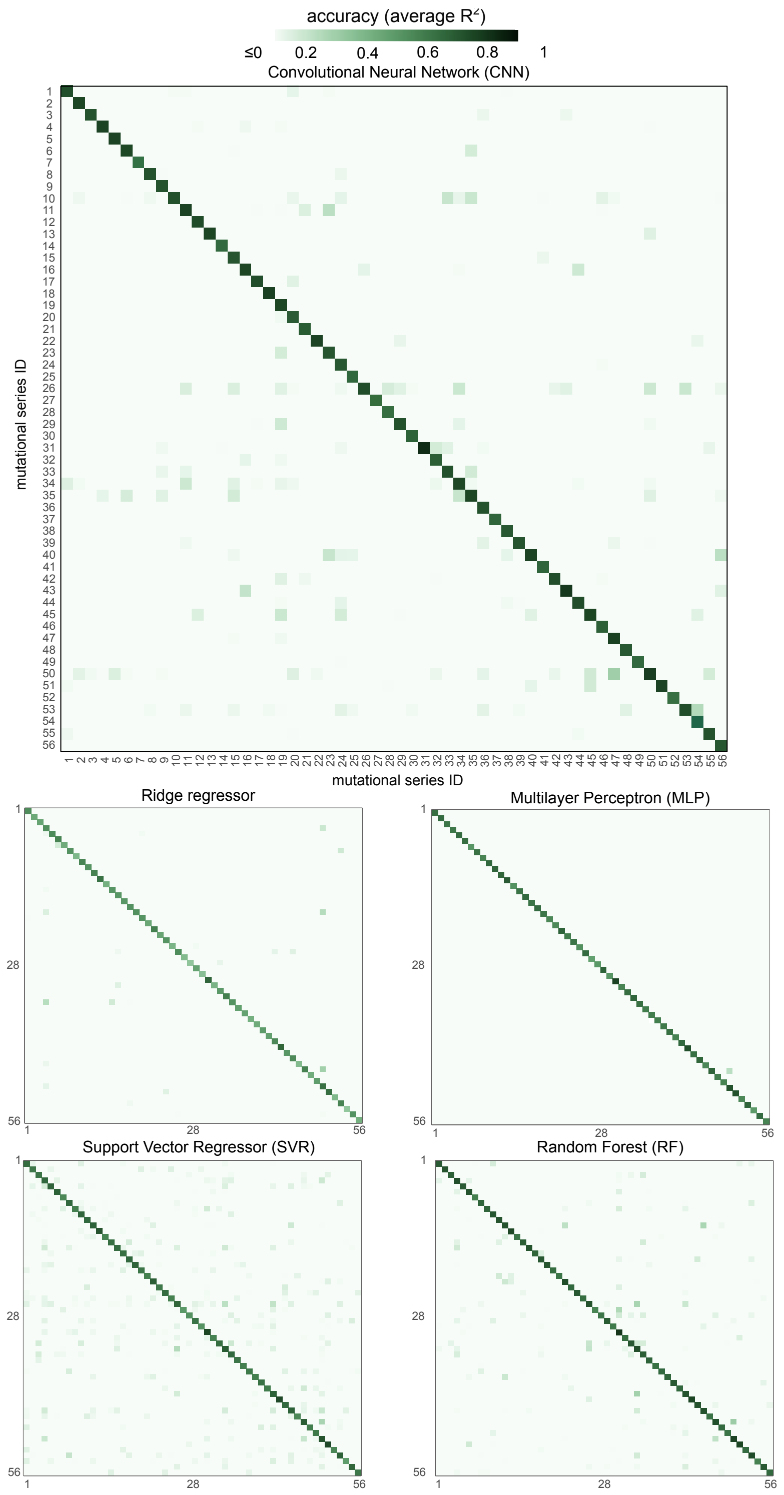

Supplementary Figure S11. **Generalization performance of machine learning models trained on a single mutational series.** Heatmaps show the accuracy (R^2) of CNNs trained on a single series and tested in all other series. Models were trained on 75% of a single mutational series and tested on held-out sequences from every other series (10% of each series). Values in the diagonal are the accuracy when tested on 10% of held-out sequences from the same series employed for training. Accuracy is reported as the R^2 computed on a held-out test set and averaged across five training repeats. The results indicate that model generalization is extremely poor, with all models achieving low or negative cross-series accuracy.

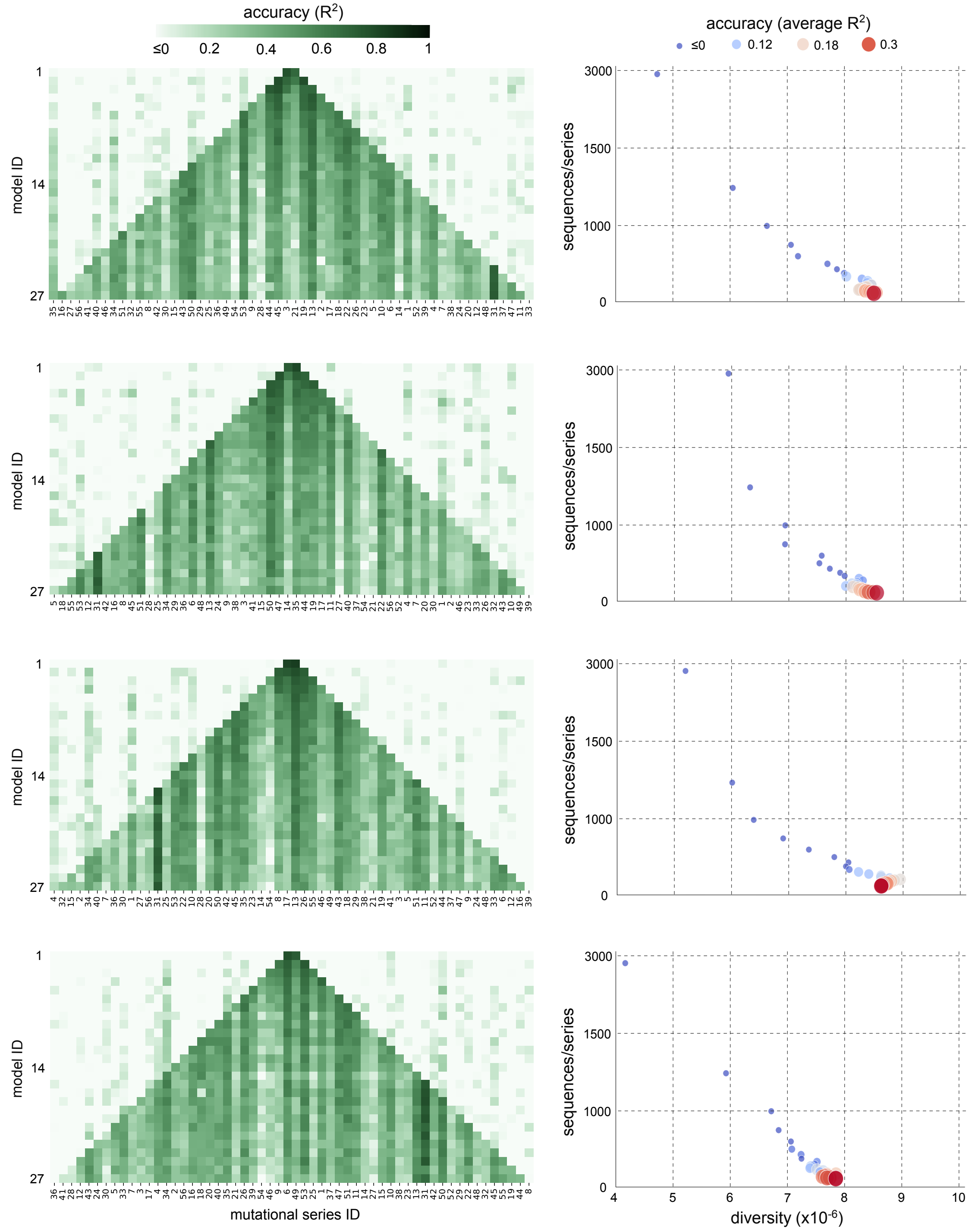

Supplementary Figure S12. **Convolutional neural networks trained on an increasingly diverse sequence space.** Shown are four repeats of the computational experiment shown in Figure 5, with randomized selection of mutational series employed for training.

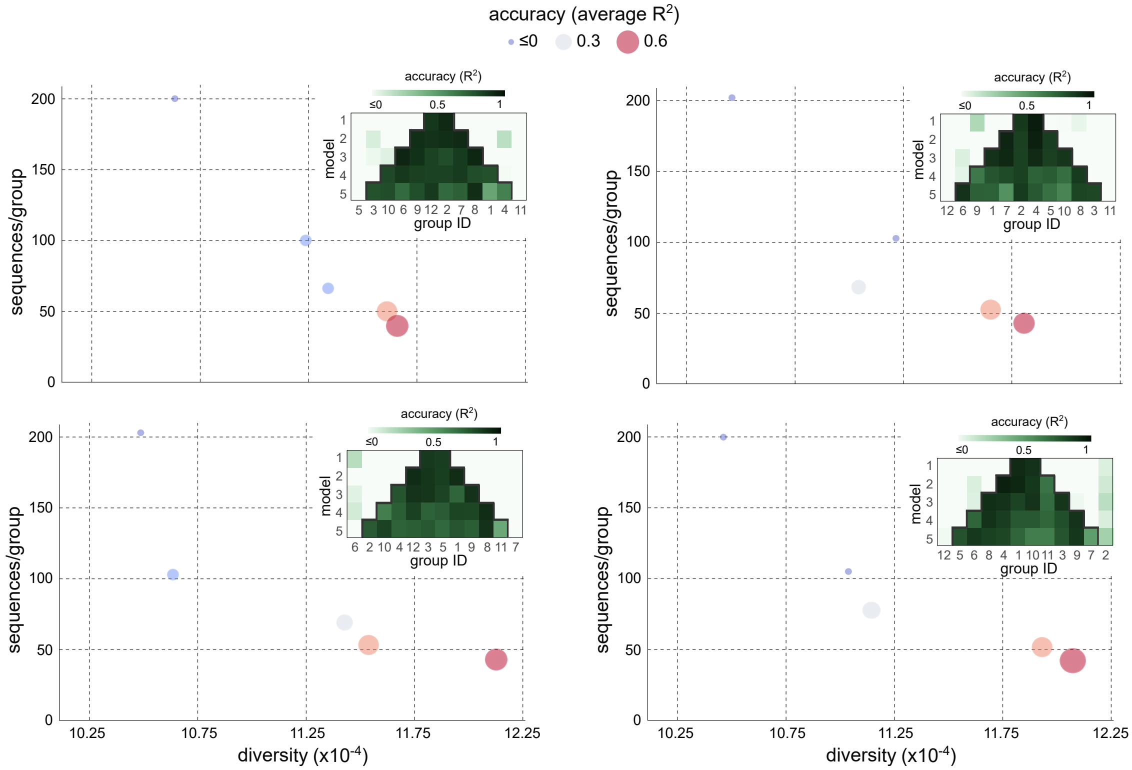

Supplementary Figure S13. **Random forests trained on an increasingly diverse sequence space for *Saccharomyces cerevisiae*.** Shown are four repeats of the computational experiment shown in Figure 6, with randomized selection of groups employed for training.

Supplementary Tables

Reference	Construct length	No. of variants
Cuperus et al, 2017[7]	50nt	500,000
Höllerer et al, 2020[8]	17nt	2,500–248,000
Angenent-Mari et al, 2020[9]	145nt	90,000
Kotopka et al, 2020[10]	246–312nt	1,000,000
de Boer et al, 2020[2]	80nt	100,000,000
Vaishnav et al, 2022[1]	80nt	20,000,000

Supplementary Table S1. **Sequence-to-expression machine learning models.** The list focuses on studies on prediction from short DNA or RNA sequences. The list excludes studies focused on whole gene prediction[11] or those that focus on other phenotypes beyond protein expression, such as transcription factor binding[12] or mRNA levels[13]; data sizes have been rounded.

**Regressor**	**Hyperparameter**	**Value**
support vector regressor	regularization (C)	$[1, 50]$
	margin tolerance (ϵ)	$[0.1, 2]$
multilayer perceptron	activation function	{ReLU, tanH}
	hidden layers	$[1, 5]$
	no. of neurons	$[100, 400]$ incr. of 50
random forest	no. of estimators	$[5, 100]$ incr. of 10
	maximum depth	$[15, 100]$ incr. of 5
	min samples per leaf	$[1, 12]$
	min samples to split	$[2, 12]$

Supplementary Table S2. **Search space for hyperparameters for non-deep models in Figure 2.** We performed an exhaustive grid-search with 10-fold cross-validation over the specified parameter values of each regressor, for all encodings.

**Regressor**	**Hyperparameter**	**Value**
ridge regressor	regularization (α)	$[10^{-1}, 10^{2}]$
support vector regressor	kernel method	RBF
	regularization (C)	30
	margin tolerance (ϵ)	0.5
multilayer perceptron	activation function	ReLU
	hidden layers	3
	no. of neurons	100
random forest	no. of estimators	25
	maximum depth	30
	min samples per leaf	3
	min samples to split	2

Supplementary Table S3. **Hyperparameters for non-deep machine learning regressors.** We employed the same hyperparameters for all combinations of mutational series and DNA encodings in all models, except the ridge regressor. The regularization strength of the ridge regressor was optimized on a case-by-case basis in the range shown.

Blocks	Hyperparameter	Range
Convolutional	number of layers	$[1, 6]$
	number of filters	$\{32, 64, 128, 256, 512\}$
	filter width	$\{3, 5, 9, 13, 15, 17, 25\}$
	dropout probability	$\{0, 0.1, 0.15, 0.2, 0.25, 0.5\}$
Dense	number of layers	$[1, 6]$
	hidden units	$\{32, 64, 128, 256, 512\}$
	dropout probability	$\{0, 0.1, 0.15, 0.2, 0.25, 0.5\}$

Supplementary Table S4. **Search space for hyperparameters of the convolutional neural network in Figure 3A.** We used subsets of the search space to run five iterations of the HyperOpt routine. In each run, HyperOpt performs Bayesian optimisation and assesses 50 combinations of hyperparameters in an informed manner.

Blocks	Hyperparameter	Value
Convolutional (1-3)	number of filters	256
	filter width	13
	dropout prob.	0.15
	activation	ReLU
	max-pooling	(2,2)
Dense (4-7)	hidden units	256
	dropout prob.	0.1
	activation	ReLU
Dense (final)	unit	1

Supplementary Table S5. **Architecture of the convolutional neural network.** For fair comparisons across datasets, we used the same architecture and hyperparameters in all CNNs. We employed 2D convolutions, without skip connections, and set the padding option to *same* for all layers, to ensure that all parts of the sequences are equally employed for training. We also included a max pooling layer to reduce the number of trainable parameters.

**Hyperparameter**	**Search space**	**Chosen value**
number of estimators	$[5, 100]$ incr. of 10	50
maximum depth	$[15, 100]$ incr. of 5	30
min samples per leaf	$[1, 12]$	3
min samples to split	$[2, 12]$	4

Supplementary Table S6. **Hyperparameter tuning for random forest regressor trained on *S. cerevisiae* promoter data[1].** We used the same search space for the random forest as in Supplementary Table S2 and performed an exhaustive grid-search with 10-fold cross-validation with one-hot encoding. We employed the same hyperparameter values for all random forest models in Figure 6B.

REFERENCES

[1]Vaishnav, E. D. *et al.* The evolution, evolvability and engineering of gene regulatory DNA. *Nature 2022 603:7901* **603**, 455–463 (2022).

[2]de Boer, C. G. *et al.* Deciphering eukaryotic gene-regulatory logic with 100 million random promoters. *Nature biotechnology* **38**, 56–65 (2020).

[3]Erb, I. & Van Nimwegen, E. Transcription factor binding site positioning in yeast: proximal promoter motifs characterize tata-less promoters. *PLoS One* **6**, e24279 (2011).

[4]Cambray, G., Guimaraes, J. C. & Arkin, A. P. Evaluation of 244,000 synthetic sequences reveals design principles to optimize translation in escherichia coli. *Nature biotechnology* **36**, 1005 (2018).

[5]Kingma, D. P. & Ba, J. Adam: A method for stochastic optimization. *arXiv preprint arXiv:1412.6980* (2014).

[6]Shrikumar, A., Greenside, P. & Kundaje, A. Learning important features through propagating activation differences. In *Proceedings of the 34th International Conference on Machine Learning - Volume 70*, 3145–3153 (JMLR.org, 2017).

[7]Cuperus, J. T. *et al.* Deep learning of the regulatory grammar of yeast 5' untranslated regions from 500,000 random sequences. *Genome research* **27**, 2015–2024 (2017).

[8]Höllerer, S. *et al.* Large-scale DNA-based phenotypic recording and deep learning enable highly accurate sequence-function mapping. *Nature communications* **11**, 1–15 (2020).

[9]Angenent-Mari, N. M., Garruss, A. S., Soenksen, L. R., Church, G. & Collins, J. J. A deep learning approach to programmable rna switches. *Nature communications* **11**, 1–12 (2020).

[10]Kotopka, B. J. & Smolke, C. D. Model-driven generation of artificial yeast promoters. *Nature Communications* **11** (2020).

[11]Avsec, Ž. *et al.* Effective gene expression prediction from sequence by integrating long-range interactions. *Nature Methods 2021 18:10* **18**, 1196–1203 (2021).

[12]Alipanahi, B., Delong, A., Weirauch, M. T. & Frey, B. J. Predicting the sequence specificities of dna-and rna-binding proteins by deep learning. *Nature biotechnology* **33**, 831–838 (2015).

[13]Agarwal, V. & Shendure, J. Predicting mrna abundance directly from genomic sequence using deep convolutional neural networks. *Cell Reports* **31** (2020).
