# Supplementary material for: Accuracy and data efficiency in deep learning models of protein expression
Source: Nat Commun. 2022 Dec 15;13:7755. doi: 10.1038/s41467-022-34902-5 (PMC9751117; doi:10.1038/s41467-022-34902-5)
Supplement: Supplementary file 2 — Reporting Summary [file 41467_2022_34902_MOESM2_ESM.pdf]

nature portfolio

Corresponding author(s): Diego Oyarzun

Last updated by author(s): Aug 21, 2022

Reporting Summary

Nature Portfolio wishes to improve the reproducibility of the work that we publish. This form provides structure for consistency and transparency in reporting. For further information on Nature Portfolio policies, see our Editorial Policies and the Editorial Policy Checklist.

Statistics

For all statistical analyses, confirm that the following items are present in the figure legend, table legend, main text, or Methods section.

n/a	Confirmed	
☐	☒	The exact sample size (*n*) for each experimental group/condition, given as a discrete number and unit of measurement
☐	☒	A statement on whether measurements were taken from distinct samples or whether the same sample was measured repeatedly
☒	☐	The statistical test(s) used AND whether they are one- or two-sided<br>*Only common tests should be described solely by name; describe more complex techniques in the Methods section.*
☒	☐	A description of all covariates tested
☒	☐	A description of any assumptions or corrections, such as tests of normality and adjustment for multiple comparisons
☐	☒	A full description of the statistical parameters including central tendency (e.g. means) or other basic estimates (e.g. regression coefficient) AND variation (e.g. standard deviation) or associated estimates of uncertainty (e.g. confidence intervals)
☒	☐	For null hypothesis testing, the test statistic (e.g. *F*, *t*, *r*) with confidence intervals, effect sizes, degrees of freedom and *P* value noted<br>*Give P values as exact values whenever suitable.*
☒	☐	For Bayesian analysis, information on the choice of priors and Markov chain Monte Carlo settings
☒	☐	For hierarchical and complex designs, identification of the appropriate level for tests and full reporting of outcomes
☐	☒	Estimates of effect sizes (e.g. Cohen's *d*, Pearson's *r*), indicating how they were calculated

Our web collection on statistics for biologists contains articles on many of the points above.

Software and code

Policy information about availability of computer code

Data collection	No software was used for data collection.
Data analysis	Python code for model training, data analysis, and data plotting can be run from Google Colaboratory and is available in Zenodo at https://doi.org/10.5281/zenodo.7273952. The code has been commented and accompanied by readme files with instructions for users to run it. We opted to use Google Colaboratory to avoid problems for users with different OS or Python distributions The code is entirely self-contained and users should not need to install any packages or dependencies in their own machines.<br><br>To ensure reproducibility, we have also provided all pre-trained CNNs and test sets, as well as code for users to re-train their own models.<br><br>Package list: typing-extensions (4.1.1), pikle (4.0), pytest (3.6.4), more-itertools (9.0.0), numpy (1.21.6), pandas 1.3.5, scipy1.7.3, sklearn1.0.2, umap-learn 0.5.3, h5py (2.10.0, 3.1.0), pyyaml (6.0), keras (2.9.0), tensorflow (2.9.2), hyperopt (0.2), deeplift (0.6.13.0), seaborn (0.11.2), matplotlib (3.2.2), verstack (3.2.4).

For manuscripts utilizing custom algorithms or software that are central to the research but not yet described in published literature, software must be made available to editors and reviewers. We strongly encourage code deposition in a community repository (e.g. GitHub). See the Nature Portfolio guidelines for submitting code & software for further information.

Data

Policy information about availability of data

All manuscripts must include a data availability statement. This statement should provide the following information, where applicable:

- Accession codes, unique identifiers, or web links for publicly available datasets
- A description of any restrictions on data availability
- For clinical datasets or third party data, please ensure that the statement adheres to our policy

This study employed two published datasets:

1) Cambray et al, Nature Biotechnology, 2018: 228,000 UTR sequences 96nt long transformed into E. coli, plus normalized sfGFP fluorescence measurements. For model training, we employed fluorescence averaged across 4 repeats from the original study.

2) Vaishnav et al, Nature, 2022: 3,929 promoter sequences 80nt long from S. cerevisiae, plus normalized YFP fluorescence measurements. For model training, we employed the singlicate data published in the original study (Supplementary Figure 4F in Vaishnav et al).

Both datasets have been cleaned and reorganized in a form suitable for machine learning analyses; the cleaned datasets are available in Zenodo at https://doi.org/10.5281/zenodo.7273952.

Field-specific reporting

Please select the one below that is the best fit for your research. If you are not sure, read the appropriate sections before making your selection.

[x] Life sciences [ ] Behavioural & social sciences [ ] Ecological, evolutionary & environmental sciences

For a reference copy of the document with all sections, see nature.com/documents/nr-reporting-summary-flat.pdf

Life sciences study design

All studies must disclose on these points even when the disclosure is negative.

Sample size

1) Cambray et al, Nature Biotechnology, 2018: The dataset (~228k sequences) was first split into 56 groups according to the two dimensional projection in Fig 1B and the original source Cambray et al, Nature Biotechnology, 2018. Within each group, we sampled sequences stratified according to the measured sfGFP fluorescence. These smaller datasets were used to train the machine learning models. We used training sets of increasing size up to N=3,000 sequences for results in Fig 2B, 3C-D, 4A-C; N = {53,480; 106,960; 160,400} for Fig 3B; and N=5,800 for the deep learning model in Fig 5.

2) Vaishnav et al, Nature, 2022: The data (3,929 sequences) has an inherent cluster structure (Figure 6A). The models in Figure 6B were trained on a dataset of fixed size (N=400 sequences). These training sets were constructed by first aggregating clusters into twelve groups containing ~320 sequences/group , and then successively merging fractions of those groups to create new training sets with improved diversity. The specific groups for the aggregates were randomly chosen from a uniform distribution.

Data exclusions

1) The original dataset in Cambray et al, Nature Biotechnology, 2018, contains ~244,000 sequences. We excluded all sequences for which there were no measurements of sfGFP fluorescence and growth rate. This reduced the dataset to ~228,000 sequences. This has been carefully explained in the Methods section.

2) The dataset from Vaishnav et al, Nature, 2022, was employed as is. No exclusions were made.

Replication

Model accuracy was reported as the coefficient of determination (R^2) between measured and predicted fluorescence. In all figures, the report R^2 is the average across the five training repeats computed on a test set; in each repeat we resampled both training and test set to obtain robust performance scores. This has been explained in the Methods section and figure captions.

Randomization

Data for model training, validation and testing were randomly sampled from the full dataset.

Blinding

When training models, investigators were blind to the test sets employed for computing model accuracy, and these were held-out early in our pipeline to avoid data leakage. Details on our strategy for holding-out test data and creating the validation data have been included in the Methods and the schematic in Supplementary Figure S3A.

Reporting for specific materials, systems and methods

We require information from authors about some types of materials, experimental systems and methods used in many studies. Here, indicate whether each material, system or method listed is relevant to your study. If you are not sure if a list item applies to your research, read the appropriate section before selecting a response.

Materials & experimental systems

n/a	Involved in the study
☒	☐ Antibodies
☒	☐ Eukaryotic cell lines
☒	☐ Palaeontology and archaeology
☒	☐ Animals and other organisms
☒	☐ Human research participants
☒	☐ Clinical data
☒	☐ Dual use research of concern

Methods

n/a	Involved in the study
☒	☐ ChIP-seq
☒	☐ Flow cytometry
☒	☐ MRI-based neuroimaging
